# Is cultural context the crucial touch? Neurophysiological and self-reported responses to affective touch in women in South Africa and the United Kingdom

**DOI:** 10.1093/scan/nsaf082

**Published:** 2025-08-11

**Authors:** Danielle Hewitt, Sahba Besharati, Victoria Williams, Michelle Leal, Francis McGlone, Andrej Stancak, Jessica Henderson, Charlotte Krahé

**Affiliations:** Department of Psychology, Institute of Population Health, University of Liverpool, Liverpool, L69 7ZA, United Kingdom; Wellcome Centre for Integrative Neuroimaging, Nuffield Department of Clinical Neurosciences, University of Oxford, Oxford, OX3 9DU, United Kingdom; Department of Psychology, School of Human and Community Development, University of the Witwatersrand, Johannesburg, 2050, South Africa; Department of Biomedical Sciences, University of Sassari, Sassari, 07100, Italy; Department of Psychology, School of Human and Community Development, University of the Witwatersrand, Johannesburg, 2050, South Africa; School of Computer Science and Applied Mathematics, Faculty of Science, University of the Witwatersrand, Johannesburg, 2050, South Africa; Department of Psychology, School of Human and Community Development, University of the Witwatersrand, Johannesburg, 2050, South Africa; SAMRC/Wits Developmental Pathways for Health Research Unit, Department of Paediatrics, Faculty of Health Sciences, School of Clinical Medicine, University of the Witwatersrand, Johannesburg, 2050, South Africa; Department of Life Sciences, Faculty of Science & Engineering, Manchester Metropolitan University, Manchester, M1 5GD, United Kingdom; Department of Psychology, Institute of Population Health, University of Liverpool, Liverpool, L69 7ZA, United Kingdom; Department of Psychology, Institute of Population Health, University of Liverpool, Liverpool, L69 7ZA, United Kingdom; Department of Primary Care and Mental Health, Institute of Population Health, University of Liverpool, Liverpool, L69 3GF, United Kingdom; School of Psychology, Liverpool John Moores University, Liverpool, L3 3AF, United Kingdom

**Keywords:** affective touch, cross-cultural research, electroencephalography, neural oscillations, attachment style

## Abstract

Affective touch, involving touch-sensitive C-tactile (CT) afferent nerve fibres, is integral to human development and well-being. Despite presumed cultural differences, affective touch research typically includes ‘Western’, minority-world contexts, with findings extrapolated cross-culturally. We report the first cross-cultural study to experimentally investigate subjective and neurophysiological correlates of affective touch in women in South Africa (SA) and the United Kingdom (UK) using (i) touch ratings and (ii) cortical oscillations for slow, CT-optimal (vs. faster, CT-suboptimal) touch on two body regions (arm and palm). We also controlled for individual differences in touch experiences, attitudes, and attachment style. Cultural context modulated affective touch: SA (vs. UK) participants rated touch as more positive and less intense, with enhanced differentiation in sensorimotor beta band oscillations, especially during palm touch. UK participants differentiated between stroking speeds, with opposite directions of effects at the arm and palm for frontal theta oscillations. Alpha band power showed consistent effects across countries. Results highlight the importance of cultural context in the subjective experience and neural processing of affective touch. Findings suggest that palm touch may hold greater social or emotional significance in SA than in the UK. Future research should further explore potential cultural influences on the meaning and function of touch across contexts.

## Introduction

Our sense of touch is essential for physical and social interaction. Discriminative touch identifies object properties and guides motor behaviour (McGlone *et al.* 2014), whereas affective touch—typically gentle, dynamic touch—fulfils affiliative (Morrison *et al.* 2010) and communicative (Kirsch *et al.* 2018, McIntyre *et al.* 2022, Krahé *et al.* 2024) social functions. Affective touch is typically associated with pleasant feelings (Löken *et al.* 2009; although see e.g. Strauss et al. 2019) and is linked to approach tendencies (Pawling *et al.* 2017). Research has charted the importance of affective touch, and more broadly, prosocial/affectionate touch, in human social development (Bendas and Croy 2021), emotion regulation (Fotopoulou *et al.* 2022), and psychological and physical well-being (Field 2010, Jakubiak and Feeney 2017). However, empirical studies on the perception and functions of affective touch have overwhelmingly been carried out in ‘Western’ contexts (sometimes termed minority-world settings, differentiating these from majority-world contexts, where most of the world’s population resides; Draper *et al.* 2023), limiting our understanding of cross-cultural variations in touch perception and processing.

Affective touch perception integrates ‘bottom-up’ peripheral afferent pathways and top-down psychological and contextual factors. Tactile stimulation activates cutaneous low-threshold mechanoreceptors via myelinated Aβ afferent fibres, resulting in rapid central processing through the somatosensory system (Abraira and Ginty 2013). Additionally, gentle stroking of hairy skin at velocities of 1–10 cms^−1^, optimally at 3 cms^−1^, preferentially activates unmyelinated C-tactile (CT) afferents (Löken *et al.* 2009), with such activation positively correlated with perceived pleasantness (Löken *et al.* 2009, Perini *et al.* 2015). Experimentally, this type of touch is commonly contrasted with faster, CT-suboptimal touch to the hairy skin (Löken *et al.* 2009) or non-hairy (glabrous) body regions such as the palm (where CT fibres are not—or only sparsely—present) to isolate the contribution of CT-fibre activation and bottom-up from top-down effects on touch perception.

Neuroimaging studies have highlighted a distributed network of brain regions involved in touch processing, including somatosensory, insular, posterior parietal, and orbitofrontal cortices ­(Morrison 2016a). Meta-analytic evidence from functional magnetic resonance imaging (fMRI) studies suggests a functional dissociation between discriminative and affective aspects of touch (McGlone *et al.* 2012). While discriminative touch is associated with greater activation of primary somatosensory cortices, affective touch is linked to stronger activation of the dorsal posterior insula, a key region for interoception (Craig 2002, 2009, Feldman *et al.* 2024) and emotional processing (Duerden *et al.* 2013). However, fMRI measures neural activity indirectly via haemodynamic responses and lacks the temporal resolution necessary to capture the rapid neural processing of tactile stimulation. By contrast, electroencephalography (EEG) provides a direct measure of neural activity with millisecond-level temporal precision, making it ideally suited to track the real-time cortical processing of dynamic touch.

EEG detects fluctuations in cortical oscillatory activity linked to sensory and affective processing. Event-related desynchronization (ERD) and synchronization (ERS) in alpha (8–13 Hz) and beta bands (16–24 Hz) are linked with cortical activation (ERD) or active inhibition (ERS) in the sensorimotor system (Pfurtscheller 1977, Pfurtscheller and Aranibar 1977). Tactile brushing stimulation of glabrous and hairy skin elicits ERD in alpha and beta bands over bilateral sensorimotor cortices, suggesting their involvement in bottom-up sensory processing and motor preparation (Gaetz and Cheyne 2006). By contrast, midfrontal theta (4–7 Hz) oscillations are implicated in top-down functions of cognitive control (Cavanagh and Frank 2014) and emotion regulation (Ertl *et al.* 2013), with increased theta power associated with more cognitively demanding or emotionally arousing stimuli. However, this is most often the case under uncertainty, anxiety, or negative emotional stimuli (Cavanagh and Shackman 2015). Conversely, CT-optimal touch has been hypothesized to promote soothing, affiliative states, with considerable evidence that it reduces stress and arousal (Walker *et al.* 2022, Kidd *et al.* 2023) and serves as a buffer in stressful situations (Morrison 2016b, von Mohr *et al.* 2017). This aligns with the idea that social touch regulates affect through homeostatic and allostatic mechanisms (Fotopoulou *et al.* 2022). Notably, CT-optimal touch (to hairy skin of the forearm) has been shown to attenuate widespread theta and parietal beta oscillations compared to CT-suboptimal touch to the same region (von Mohr *et al.* 2018a), potentially reflecting a soothing, regulatory response. Individual differences further shape these dynamics: e.g. hand-holding after negative affect induction attenuates theta activity, but only in people with a secure attachment style (Kraus *et al.* 2020), underscoring the role of social-emotional context in shaping neural responses to touch.

Psychological factors and individual differences influence the perception and meaning (Sailer and Leknes 2022) as well as the neurophysiological correlates (Haggarty *et al.* 2020, Kraus *et al.* 2020) of affective touch. Touch exposure (Sailer and Ackerley 2019) and attitudes (e.g. as part of attachment styles; Krahé *et al.* 2018) modulate perceived affective touch pleasantness. Moreover, sex and gender effects are apparent, with females rating affective touch more positively (Russo *et al.* 2020) and women ascribing different meanings to touch (Krahé *et al.* 2024) compared to males/men. At a cultural level, individuals from collectivist cultures report higher acceptability of affectionate touch (Burleson *et al.* 2019, Sorokowska *et al.* 2021), but the few studies investigating the role of culture in affective touch (Burleson *et al.* 2019, Suvilehto *et al.* 2019, Sorokowska *et al.* 2021, Schirmer *et al.* 2023) have primarily focused on self-reported outcomes, with no studies on the neurophysiological correlates of affective touch in different cultural contexts. In the present study, we focused on comparing the United Kingdom (UK) and South Africa (SA). SA shares features associated with more positive touch norms and greater touch frequency, such as stronger collectivist tendencies and a warmer climate (Sorokowska *et al.* 2021). Cultural variations in touch norms (Burleson *et al.* 2019) and early touch experiences (e.g. baby wearing and co-sleeping; Schön and Silvén 2007), together with the influence of ‘top-down’ factors on touch perception and evaluation (Sailer and Leknes 2022), suggest that the neural processing of affective touch may differ in SA vs. UK cultural contexts, but this has not yet been explored.

Accordingly, this pre-registered experimental study conducted in the UK and SA explored how cultural context shapes touch evaluations and neural oscillations (captured using EEG) during affective touch. Using a within-subjects design, we varied touch velocity (affective, i.e. slow, CT-optimal, vs. faster, CT-suboptimal) and body region (arm vs. palm) to tease apart the influence of bottom-up vs. top-down effects, whilst controlling for individual differences in touch experiences, attitudes, and attachment styles. As noted in our pre-registration, hypotheses were exploratory in nature. We tentatively hypothesized that SA participants would evaluate affective touch more positively and, given potentially greater touch exposure (e.g. Sailer and Ackerley 2019) in the SA context, that SA participants would show enhanced differentiation (to slow vs. faster touch) in neural oscillations compared to UK participants. More broadly across cultural contexts, we hypothesized that slower-velocity, affective touch would be evaluated more positively than faster-velocity touch (Löken *et al.* 2009) and would be associated with decreased theta band activity (specifically, increased ERD) in response to CT-optimal touch (cf. von Mohr *et al.* 2018a). We also examined the effects of affective touch on alpha and beta band activity to capture sensorimotor processes. Given greater innervation of Aβ fibres and the relevance of the hand for reach-to-grasp movements, we explored whether increased alpha and beta ERD would be evident for the palm vs. the arm.

## Materials and methods

The study was pre-registered on the Open Science Framework: https://osf.io/fcqnk. Ethical approval was obtained from the Institute of Population Health Research Ethics Committee, University of Liverpool, and the Human Research Ethics Committee (Medical) at the University of the Witwatersrand. Data collection ran in the summer in each respective country: from June to August 2022 in the UK and November 2023 to January 2024 in SA. Data were not analysed until all participants had been tested.

### Design

The study employed a 2 (country: UK, SA; between-subjects) × 2 (touch velocity: slow, CT-optimal 3 cms^−1^ vs. faster, CT-suboptimal 18 cms^−1^ ; within-subjects) × 2 (body region: CT-innervated forearm vs. non-CT-innervated palm; within-subjects) mixed design. All participants received slower and faster velocity touch to the arm and palm of the hand (one block per condition), with order counterbalanced across participants, while EEG was recorded. Outcome measures were: (i) self-reported pleasantness, comfort, intensity, liking, and wanting ratings of touch; and (ii) theta, alpha, and beta neural oscillations. We also measured self-reported experiences and attitudes to touch (Trotter *et al.* 2018) and adult attachment style (Fraley *et al.* 2000).

### Participants

*N *= 36 female participants (given biological sex differences in touch perception; Russo *et al.* 2020) were recruited in two different countries: *N *= 15 in Liverpool, UK, and *N *= 21 in Johannesburg, SA. The sample size was based on previous research (*N *= 28 in the similar EEG study by von Mohr *et al.* 2018a). We exceeded our target of *N *= 30 indicated in the OSF pre-registration. EEG data from two SA participants were excluded prior to data analysis: one due to poor electrode impedances and missing behavioural data, and one due to a technical issue resulting in missing event markers. This resulted in a final EEG sample of 19 SA participants.

Participants were all aged 18 or over (*M = *23.14 years, SD* = *7.32 in SA, and *M = *25.93 years, SD* = *6.39 in UK), with no significant differences in age between countries (see Table 1 for full demographic characteristics and difference tests). All participants were right-handed, as touch was administered to the non-dominant left arm and palm. Exclusion criteria were: a history of psychiatric, neurological, or medical conditions affecting touch perception (e.g. chronic pain) and wounds, scars, tattoos, or skin conditions on the forearm or palm.

**Table 1.** Demographic characteristics and self-report questionnaires.

		South Africa (*N = *21)		United Kingdom (*N = *15)		Group comparison (uncorrected)
		Mean (SD)	Min–max	Mean (SD)	Min–max	Bootstrapped regression analysis
Age		23.14 (7.32)	18–52	25.93 (6.39)	20–38	*b = *2.79, SE* = *2.23, *p* = .211, 95% CIs: −1.58; 7.16
Number co-habitants		2.57 (1.72)	0–6	2.8 (2.4)	0–7	*b = *0.23, SE = 0.71, *p* = .747, 95% CIs: −1.16; 1.61
Religiosity		0.62 (1.5)	−2 to 2	−1.27 (0.88)	−2 to 1	*b = *−1.89, SE = 0.40, *p <* .001, 95% CIs: −2.66; −1.11
ECR-R	Attachment anxiety	3.19 (1.24)	1.33–5.94	3.26 (1.15)	1.44 to 4.83	*b = *0.07, SE = 0.39, *p* = .856, 95% CIs: −0.70; 0.84
	Attachment avoidance	2.84 (1)	1.39–5.17	2.7 (1.18)	1–5.72	*b = *−0.14, SE = 0.38, *p* = .710, 95% CIs: −0.89; 0.61
TEAQ	Family and friends touch	3.79 (0.66)	2.45–4.91	3.79 (0.93)	2.09–5	*b = *0.00, SE = 0.27, *p* = .998, 95% CIs: −0.53; 0.53
	Current intimate touch	3.33 (0.73)	1.93–4.64	3.7 (0.9)	1.64–4.86	*b = *0.37, SE = 0.28, *p* = .186, 95% CIs: –0.18; 0.93
	Childhood touch	3.82 (0.77)	2.44–5	3.62 (1.3)	1.33–5	*b = *−0.19, SE = 0.36, *p* = .587, 95% CIs: −0.89; 0.50
	Attitude to self-care	3.77 (0.83)	2.2–5	3.59 (1.19)	0.8–5	*b = *−0.19, SE = 0.34, *p* = .584, 95% CIs: −0.86; 0.48
	Attitude to intimate touch	3.98 (0.76)	2.62–4.92	4.38 (0.66)	3–5	*b = *0.40, SE = 0.23, *p* = .086, 95% CIs: −0.06; 0.85
	Attitude to unfamiliar touch	2.77 (0.83)	1.6–4.6	3.41 (0.89)	1.4–4.8	*b = *0.65, SE = 0.28, *p* = .021, 95% CIs: 0.10; 1.20
		***N* (%)**		***N* (%)**		**Chi-square test**
Ethnicity	Arab	0 (0)		1 (6.67)		Pearson *χ* ^2^(5) = 25.27, *p <* .000
	Asian	1 (4.76)		1 (6.67)		
	Black	16 (76.19)		0 (0)		
	Latin American	0 (0)		1 (6.67)		
	Mixed	3 (14.29)		2 (13.33)		
	White	1 (4.76)		10 (66.67)		
Marital status	Single	17		7		Pearson *χ* ^2^ (1) = 5.84, *p* = .016
	In a relationship	3		8		
Highest level of education	Diploma	0 (0)		1 (6.67)		Pearson *χ* ^2^ (3) = 16.36, *p* = .001
	High school	17 (80.95)		2 (13.33)		
	Postgraduate degree	2 (9.52)		7 (46.67)		
	Undergraduate degree	2 (9.52)		5 (33.33)		

Religiosity: Participants responded to the item “How much do you agree with the following statement? I am very religious” on a scale from -2 (‘strongly disagree’) to 2 (‘strongly agree’).

ECR-R, Experiences in Close Relationships Revised Questionnaire; TEAQ, Touch Experiences and Attitudes Questionnaire.

Marital status was ‘divorced’ for one SA participant, not included here as unclear whether or not they were single. All participants reported being biologically female and self-identified as women.

### Materials and measures

#### Touch protocol

Touch was administered by a trained experimenter unknown to participants, using a cosmetic make-up brush (Natural Hair Blush Brush, No. 7, The Boots Company). Four 9 × 4 cm areas were marked on the participant’s skin: two contiguously along the participant’s left volar forearm between wrist and elbow, and two side-by-side on the surface of the palm. Touch was administered in a block design across four conditions (order counterbalanced, with one block per condition): CT-innervated arm at 3 cms^−1^, CT-innervated arm at 18 cms^−1^, non-CT-innervated palm at 3 cms^−1^, and non-CT-innervated palm at 18 cms^−1^, as in previous research contrasting CT-optimal and non-CT-optimal touch (e.g. Krahé *et al.* 2016, von Mohr *et al.* 2018b, Meijer *et al.* 2024). For the 3 cms^−1^ condition, a single brush stroke was delivered manually from proximal to distal within the marked area. For the 18 cms^−1^ condition, six proximal-to-distal strokes were delivered in the same region. CT afferents show an inverted U-shaped response to dynamic stimuli, with firing rates broadly tuned to stimuli between 1 and 10 cms^−1^, and maximal firing at 3 cms^−1^. Microneurography studies have shown that stroking at higher speeds elicits very few CT responses (Löken *et al.* 2009, Ackerley *et al.* 2014b). Therefore, minor variations in brushing speed are highly unlikely to impact neural responses if they are in the correct CT-optimal or non-CT-optimal ranges, as was the case here.

Each block included 5 practice trials (not included in analyses) and 40 3 s trials, separated by 8 s of no touch. Adjacent skin areas were alternated between strokes to prevent habituation. Touch onsets were synchronized with a 4-s auditory countdown, audible only to the experimenter through headphones. The use of audio or visual cues to trigger manual brushing is well established in affective touch research (e.g. Björnsdotter *et al.* 2009, Morrison *et al.* 2011, Lucas *et al.* 2015, Haggarty *et al.* 2020).

To ensure consistency in touch administration, each experimenter was trained using a standardized video and followed a protocol aligned across sites. Only one experimenter administered touch in each site. A virtual pilot session was conducted with each site to observe and verify adherence to the touch and EEG protocol. Brushing speed and duration were maintained through internal pacing and extensive training, while consistent pressure was ensured by maintaining full brush contact across the entire marked region throughout strokes. Participants provided single ratings for pleasantness, comfort, intensity, liking, and wanting ratings of touch after each block on visual analogue scales with the anchors 0 (‘not at all’) to 100 (‘extremely’).

#### Electroencephalography

EEG data were recorded from 64 active silver–silver chloride electrodes using a BrainProducts actiCap snap system (BrainProducts GmbH, Munich, Germany) in the UK, and a g.tec g.Hlamp (g.tec medical engineering GmbH, Schiedlberg, Austria) in SA. Electrodes were embedded in a cap, positioned in line with anatomical landmarks according to the international 10–20 system. The BrainProducts actiCap system utilized Fz as the reference electrode and FPz as the ground electrode (63 active recording electrodes). The g.tec system utilized linked earlobe references A1 and A2 and AFz as the ground electrode (62 active recording electrodes). Electrode-to-skin impedances were kept below 5 kΩ for g.tec and 25 kΩ for BrainProducts. Signals were digitized at 1 kHz using an actiChamp (UK) or g.Hlamp (SA) DC amplifier and stored for offline analysis.

#### EEG data processing

EEG data were processed using EEGLab (Delorme and Makeig 2004). Continuous data were split into 8-s epochs (−2.5 to 5.5 s around touch onset) and combined into one data file for each participant. Data were re-referenced to the common average. Original reference channels were not regenerated to maintain consistency across sites, as different reference channels were used for the UK and SA datasets. Data were filtered using 1 Hz high-pass and 70 Hz low-pass filters. A notch filter from 48 to 52 Hz was applied to remove mains line noise before downsampling to 256 Hz.

Artefacts were removed using a semi-automated method in EEGLab (see Supplementary Materials for details). Power spectra were computed in FieldTrip (http://fieldtriptoolbox.org) using a discrete Fourier time–frequency transformation. Power spectral densities were computed in the 8-s epochs (−2.5 to 5.5 s around touch onset) using Welch’s method from 1-s overlapping segments. Data were smoothed using a 4 Hz Slepian sequence prior to the Fourier transformation. The spectral window was shifted in 0.1 s intervals to yield a power time series of 80 points. Spectral power was estimated in the range 1–70 Hz with a frequency resolution of 1 Hz. Relative power was evaluated using the classical ERD transformation (Pfurtscheller and Aranibar 1977): $D\%=(100*\;\frac{A-R}{R}),\;$ where D represents the percentage power change during epochs following stimulus onset (A, 0 to 5.5 s) relative to baseline (R, −2 to −1 s). Positive D values correspond to relative power decreases (ERD), while negative D values correspond to power increases (ERS; Pfurtscheller 1977, Pfurtscheller and Aranibar 1977).

#### Questionnaires

##### Experiences in Close Relationships Revised questionnaire (ECR-R; Fraley *et al.* 2000)

The ECR-R assesses individual differences in attachment anxiety and avoidance in adult romantic relationships. It consists of 36 items rated on a 7-point Likert scale, where 1 = ‘strongly disagree’ and 7 = ‘strongly agree’, and produces separate scores for attachment anxiety and attachment avoidance, with higher scores denoting greater anxiety/avoidance. Cronbach’s alphas were α = .93 for both anxiety and avoidance in the current sample (across countries).

##### Touch Experiences and Attitudes Questionnaire (TEAQ; Trotter *et al.* 2018)

The TEAQ includes 57 items scored on a 5-point Likert-type scale (1 =‘strongly’ disagree to 5 = ‘strongly agree’), with a mean score calculated for each of six subscales: friends and family touch (FFT), current intimate touch (CIT), childhood touch (ChT), attitude to self-care (ASC), attitude to intimate touch (AIT), and attitude to unfamiliar touch (AUT). Higher scores denote more positive attitudes and experiences. The TEAQ has recently been validated in an SA sample (Puckle 2021). Cronbach’s alphas were α = .84 for FFT, α = .88 for CIT, α = .92 for ChT, α = .77 for ASC, α = .89 for AIT, and α = .74 for AUT.

#### Procedure

**Figure 1.**
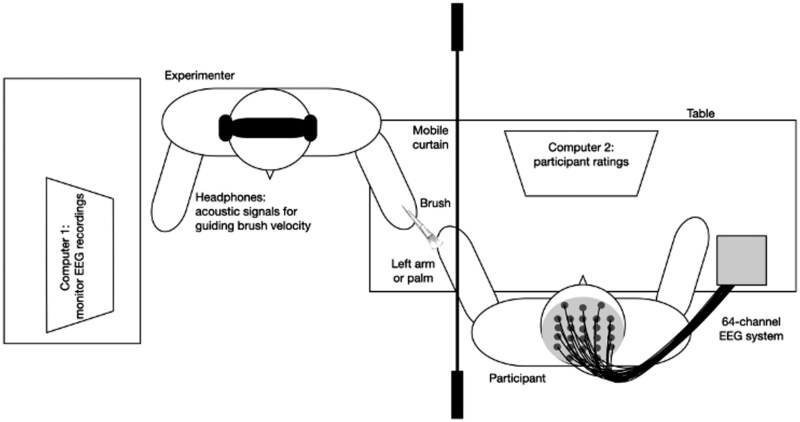
The schema depicts the experimental set-up in South Africa and in the United Kingdom. Slight alterations in the room set-up were made due to constraints on the physical laboratory space, but the main layout and materials used were standardized and consistent across both study sites.

Participants attended one laboratory visit (see Fig. 1 for setup). After obtaining informed consent, the EEG cap was fitted by a female researcher while participants completed the questionnaires. Thereafter, touch blocks were administered while EEG data were recorded. During these blocks, a screen was placed to prevent participants from seeing the experimenter and tactile stimulation. After all blocks were completed, the EEG cap was removed by the researcher, and participants were fully debriefed and compensated for their time.

### Statistical analyses

The analysis plan was pre-registered, with deviations noted below as they appear.

Amplitude changes of cortical oscillations were examined by exporting relative power (ERD/S) in theta (4–7 Hz), alpha (8–13 Hz), and beta (16–24 Hz) frequency bands from bilateral electrode sites associated with touch processing: frontal (F1, F2, F3, F4), central (C1, C2, C3, C4, Cz), and parietal (P1, P2, P3, P4, Pz) regions for alpha and beta band power, and frontal and central clusters for theta band power, based on previous literature showing maximal changes in these bands at the respective sites for tactile stimulation (Gaetz and Cheyne 2006, Henderson *et al.* 2023, Hewitt *et al.* 2023). Bilateral clusters were pre-registered based on a previous EEG study on affective touch (von Mohr *et al.* 2018a) and well-documented prior evidence that somatosensory stimulation is followed by bilateral 10 and 20 Hz ERD over sensorimotor cortices (reviewed in Stančák 2006; also Fallon *et al.* 2013). Restricting analyses to contralateral alpha power would therefore capture only part of the neural response to affective touch, whereas bilateral analysis offers a more complete representation. The window for ERD analysis was the sustained brushing period between 0.5 and 3 s to avoid contamination of transient, non-stationary artefacts at the onset of brushing, motivated by the delayed central processing associated with slow-conducting CT afferents (Vallbo *et al.* 1993, Löken *et al.* 2009) and prior research showing that affective touch responses are more evident in later neurophysiological responses (e.g. ultralate potentials) rather than early, transient responses (Ackerley *et al.* 2013, Haggarty *et al.* 2020). Moreover, sustained theta dynamics have been documented in various studies investigating cognitive or emotional processing, including emotion regulation (Zouaoui *et al.* 2023), working memory (Raghavachari *et al.* 2001), and emotional memory retrieval (Zheng *et al.* 2019), illustrating the appropriateness of this analysis window for continuous, dynamic touch. This window also allowed us to minimize any potential variation in manually applied brushing. Post-stimulus changes (3–5.5 s) were not analysed in the current study to avoid dilution of the effects from the active touch stimulus.

Deviations from pre-registration based on an updated review of this literature included: amended frequency bands in theta (4–7 Hz; 4–8 Hz in pre-registration) and beta bands (16–24 Hz; 13–30 Hz in pre-registration); relative power was not exported from delta or gamma bands, the former being primarily associated with slow-wave sleep (e.g. reviewed in Long *et al.* 2021), while the latter is frequently produced by microsaccades and muscle activity (Yuval-Greenberg *et al.* 2008); prefrontal, temporal, and occipital regions were not included to restrict the number of comparisons; and ERPs are not reported to focus rather on sustained cortical oscillations during dynamic touch. Following EEG data cleaning, blocks with <30 trials were removed from further analysis, based on previous work (Pfurtscheller 1977). A total of eight blocks were removed across six participants (four with one excluded block and two with two excluded blocks each: full details in Supplementary Materials). The remaining valid blocks for these participants were retained in statistical analyses.

#### Hypothesis testing

Multivariate linear mixed models (MLMMs) in Stata 18 (StataCorp 2023) were used to test hypotheses. Outcomes were self-report ratings and spectral power. All data provided by participants were included for touch ratings. MLMMs can handle missing data under the assumption that data are missing at random. Where EEG data were missing entirely (in two participants, due to technical issues or persistent noise in the recording), self-report data were still included. In each analysis, we included ECR-R and TEAQ subscale scores as fixed-effect covariates. Fixed effects of interest were touch velocity (3 cms^−1^ vs. 18 cms^−1^; categorical predictor) and region (arm vs. palm; categorical predictor). Including random slopes for velocity and location did not change any of the results, so these were not included in final models. All interaction terms were included, and significant interactions were followed up with planned contrasts (Bonferroni corrected). The intercept of the participant ID was a random effect. We originally planned to include frequency band and region in analyses as fixed effects of interest. However, contrary to our pre-registered analysis plan, we instead ran analyses separately for each frequency band and region, as directly comparing frequency bands or regions was not deemed useful. To account for the increase in the number of analyses, we additionally applied Benjamini–Hochberg corrections to the results (false discovery rate set to 5%) and report in the text only those effects that survived correction; full results are in the tables. We calculated marginal *R*^2^ (representing the variance explained only by fixed effects) and conditional *R*^2^ (including fixed and random effects) for each model. Given the presence of significant interactions, we did not try to isolate *R*^2^ values for individual predictors.

#### Exploratory analyses

The above confirmatory MLMMs were re-run, adding country (UK, SA) as a between-subjects categorical predictor and examining its interactions with touch velocity and body region on outcome ratings. The country comparisons, while labelled exploratory here and in our pre-registration due to the novel and multifactorial nature of the data, were central to the study’s aims and thus are presented prominently in the Results section.

## Results

The data showed a strong modulatory effect of cultural context, such that the effects of touch velocity and location differed significantly between UK and SA participants. Given this and the centrality of cultural context to our study aims, we focus here on analyses including country (UK vs. SA) as a between-subjects factor. Confirmatory within-subject effects of touch velocity and location, derived from the affective touch literature, are reported in the Supplementary Materials.

### Descriptive statistics

Self-reported touch ratings are presented by country in Table 2 (see Supplementary Material Table 2 for ratings across countries). There were no differences between SA and the UK regarding attachment styles and experiences and attitudes to touch, except that UK participants had significantly more positive attitudes to unfamiliar touch than did SA participants.

**Table 2.** Descriptive statistics for touch ratings by country.

			South Africa						United Kingdom					
Body region	Velocity	Rating	Mean	SD	*N*	Min	Max	Range	Mean	SD	*N*	Min	Max	Range
Palm	Slow	Like	74.71	25.25	21	21	100	79	67.73	23.52	15	21	100	79
		Want	62.62	30.29	21	0	100	100	58.07	26.49	15	0	89	89
		Intense	28.14	31.55	21	0	100	100	45.80	27.61	15	0	90	90
		Comfortable	80.52	25.35	21	2	100	98	75.47	15.27	15	50	100	50
		Pleasant	79.71	21.30	21	28	100	72	75.13	17.19	15	40	100	60
	Fast	Like	76.24	22.05	21	25	100	75	55.20	26.69	15	13	100	87
		Want	65.33	27.38	21	5	100	95	46.40	23.27	15	5	78	73
		Intense	28.43	31.28	21	0	100	100	53.80	26.06	15	3	90	87
		Comfortable	82.76	20.88	21	34	100	66	66.0	19.63	15	33	100	67
		Pleasant	77.76	20.46	21	41	100	59	61.00	22.93	15	25	100	75
Arm	Slow	Like	87.10	20.10	20	16	100	84	64.80	22.31	15	21	93	72
		Want	75.95	21.57	20	29	100	71	49.40	24.14	15	0	79	79
		Intense	27.15	30.25	20	0	85	85	49.53	14.63	15	13	70	57
		Comfortable	84.65	26.10	20	8	100	92	73.60	20.75	15	30	100	70
		Pleasant	89.00	19.59	20	14	100	86	68.60	16.15	15	37	100	63
	Fast	Like	73.33	27.64	21	10	100	90	54.93	22.77	15	25	100	75
		Want	64.19	24.07	21	2	100	98	47.20	24.48	15	0	85	85
		Intense	27.81	30.09	21	0	100	100	52.13	25.99	15	0	90	90
		Comfortable	82.57	19.10	21	38	100	62	62.27	21.13	15	30	100	70
		Pleasant	77.67	19.93	21	40	100	60	60.00	18.65	15	29	100	71

### Touch ratings

The four evaluative ratings (liking, wanting, comfort, and pleasantness) formed a highly internally consistent scale (see Supplementary Materials). Therefore, our outcomes were ‘touch evaluation’ (four ratings entered concurrently into MLMMs) and touch intensity (single rating).

Model results are presented in Table 3 (marginal *R*^2^ = 0.26 and conditional *R*^2^ = 0.56 for the full touch evaluation model and marginal *R*^2^ = 0.35 and conditional *R*^2^ = 0.81 for the full intensity model). There was an effect of velocity on evaluative touch ratings that was not qualified by country or body region: participants in SA and UK evaluated affective, slow touch (*M *= 73.47, SE = 2.48) significantly more positively than faster touch (*M *= 67.34, SE = 2.47), in line with our hypothesis, though the faster touch was still evaluated moderately positively. Ratings did not differ by body region. Examining effects of country, participants in SA evaluated touch significantly more positively (*M = *76.78, SE* = *3.37) and significantly less intense (*M* = 23.63, SE = 5.00) than did UK participants (evaluative rating: *M* = 61.54, SE = 4.08; intensity rating: *M* = 56.02, SE = 6.06) across stroking velocities and body regions (see Table 3), in line with our hypothesis.

**Table 3.** Linear mixed modelling results for evaluative (multivariate) and intensity (univariate) touch rating outcomes.

		Evaluation					Intensity					
		*b*	SE	*p*	95% CIs		*b*	SE	*p*	95% CIs		
Predictors of interest	Country (SA is reference)	−18.53	6.19	**.003**	−30.66	−6.40	34.01	9.28	**<.001**	15.82	52.20	
	Velocity (fast is reference)	7.95	2.58	**.002**	2.90	13.00	−1.75	4.07	.667	−9.73	6.23	
	Country × velocity	0.05	3.95	.989	−7.70	7.80	−0.85	6.25	.892	−13.10	11.40	
	Body region (arm is reference)	1.08	2.53	.669	−3.88	6.05	0.62	4.01	.877	−7.23	8.47	
	Country × body region	−0.03	3.93	.993	−7.73	7.66	1.05	6.21	.866	−11.12	13.21	
	Velocity × body region	−9.08	3.61	.012	−16.16	−2.00	1.47	5.71	.797	−9.73	12.66	
	Country × velocity × body region	13.03	5.57	.019	2.11	23.95	−6.87	8.81	.436	−24.13	10.40	
Covariates	Attachment avoidance	1.77	3.29	.590	−4.67	8.22	2.07	4.88	.671	−7.50	11.64	
	Attachment anxiety	−8.33	2.76	**.003**	−13.74	−2.91	−7.70	4.10	.061	−15.74	0.34	
	Friends and family touch	0.94	4.39	.830	−7.66	9.54	7.21	6.51	.268	−5.55	19.98	
	Current intimate touch	−4.74	5.44	.383	−15.40	5.92	−13.59	8.07	.092	−29.41	2.24	
	Childhood touch	4.76	3.01	.114	−1.15	10.67	7.94	4.48	.076	−0.83	16.71	
	Attitude to self-care	−1.49	3.02	.623	−7.40	4.43	2.42	4.48	.589	−6.37	11.21	
	Attitude to intimate touch	5.38	7.05	.446	−8.45	19.21	3.14	10.47	.764	−17.39	23.67	
	Attitude to unfamiliar touch	2.03	3.60	.573	−5.03	9.10	−4.67	5.35	.383	−15.16	5.82	
Intercept		68.58	24.87	.006	19.84	117.33	25.40	36.94	.492	–46.99	97.79	

Bold *p* values denote those effects that survived Benjamini–Hochberg corrections.

### Electroencephalography (ERD/S)

**Figure 2.**
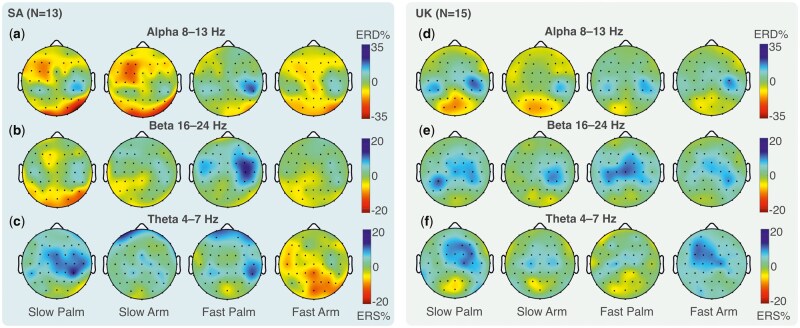
Grand-averaged event-related desynchronization/synchronization (ERD/S) during touch (0–3 s) by country in alpha (a, d), beta (b, e), and theta (c, f) bands in each of the four conditions for SA and UK participants. Only participants with data for all conditions are shown in the topographic plots (as matrices need to be equal), resulting in a slightly smaller number here than the number included in statistical analyses. Topographic plots show only electrodes that were common across countries.

Grand-averaged ERD/S in alpha, beta, and theta bands in each of the four conditions, averaged over participants within the UK and SA, are presented in Fig. 2. Cortical activation changes during dynamic touch as a function of velocity, body region, and country were evaluated using MLMMs.

#### Alpha band

No effects of country (alone or in interaction with velocity and/or body region) survived Benjamini–Hochberg corrections (see Table 4). However, there was an effect of velocity across countries at central sites (marginal *R*^2^ = 0.31 and conditional *R*^2^ = 0.69 for the full model involving central electrodes and alpha band oscillations). Alpha-band ERS was significantly greater for slow-velocity touch (*M = *−0.32, SE* = *3.43) compared to ERD for faster-velocity touch (*M = *5.41, SE* = *3.42) across body regions. There was also an effect of body region at both central and parietal alpha sites (marginal *R*^2^ = 0.22 and conditional *R*^2^ = 0.43 for the model involving parietal electrodes). Alpha-band ERS was significantly larger for the arm vs. greater ERD at the palm at central (*M = *−0.58, SE* = *3.43 for arm; *M = *5.81, SE* = *3.43 for palm) and parietal sites (*M = *−8.99, SE* = *1.72 for arm; *M = *−5.33, SE* = *1.72 for palm) across stroking speeds. Therefore, alpha band oscillations were not influenced by cultural context but were modulated by stroking speed and body region.

**Table 4.** Multivariate linear mixed modelling results for country differences in alpha, beta, and theta ERD/S.

			Central electrode sites					Parietal electrode sites				
Frequency band			*b*	SE	*p*	95% CIs		*b*	SE	*p*	95% CIs	
**Alpha**	Predictors of interest	Country (SA is reference)	9.01	8.34	.280	−7.33	25.34	8.58	4.36	.049	0.04	17.12
		Velocity (fast is reference)	−7.77	2.70	**.004**	−13.07	−2.48	2.09	2.30	.363	−2.41	6.60
		Country × velocity	1.69	3.89	.663	−5.93	9.32	−6.08	3.32	.067	−12.59	0.42
		Body region (arm is reference)	9.38	2.65	**<.001**	4.18	14.57	6.35	2.26	**.005**	1.92	10.78
		Country × body region	−9.09	3.86	.018	−16.65	−1.53	−0.59	3.29	.858	−7.04	5.86
		Velocity × body region	−2.61	3.87	.500	−10.21	4.98	−4.86	3.29	.140	−11.32	1.59
		Country × velocity × body region	10.92	5.54	.049	0.06	21.78	−0.07	4.72	.988	−9.32	9.19
	Covariates	Attachment avoidance	8.06	4.84	.096	−1.43	17.54	7.02	2.33	**.003**	2.47	11.58
		Attachment anxiety	−10.84	4.35	.013	−19.36	−2.32	−6.37	2.09	**.002**	−10.46	−2.28
		Friends and family touch	1.90	6.08	.755	−10.02	13.82	0.45	2.92	.876	−5.26	6.17
		Current intimate touch	−11.19	7.52	.137	−25.94	3.55	−1.97	3.63	.587	−9.09	5.14
		Childhood touch	−3.37	4.15	.418	−11.51	4.77	1.87	1.99	.349	−2.04	5.78
		Attitude to self-care	−6.12	4.32	.156	−14.58	2.34	0.88	2.07	.671	−3.18	4.94
		Attitude to intimate touch	2.74	10.11	.787	−17.08	22.56	2.27	4.87	.642	−7.28	11.82
		Attitude to unfamiliar touch	8.77	5.22	.093	−1.46	19.01	2.74	2.51	.274	−2.17	7.65
	Intercept		38.76	34.57	.262	−29.01	106.52	−35.62	16.75	.033	−68.45	−2.79
**Beta**	Predictors of interest	Country (SA is reference)	7.64	3.53	.031	0.72	14.56	6.46	3.54	.068	−0.48	13.40
		Velocity (fast is reference)	1.41	1.42	.323	−1.38	4.20	0.36	1.38	.794	−2.35	3.07
		Country × velocity	−2.56	2.05	.212	−6.59	1.46	−2.30	1.99	.248	−6.21	1.60
		Body region (arm is reference)	10.30	1.40	**<.001**	7.56	13.04	4.82	1.36	**<.001**	2.16	7.48
		Country × body region	−7.45	2.04	**<.001**	−11.44	−3.46	−5.54	1.97	**.005**	−9.41	−1.67
		Velocity × body region	−8.22	2.04	**<.001**	−12.23	−4.22	−6.26	1.98	**.002**	−10.14	−2.37
		Country × velocity × body region	7.07	2.92	.016	1.34	12.80	9.69	2.84	**.001**	4.14	15.25
	Covariates	Attachment avoidance	1.12	2.00	.573	−2.79	5.04	2.99	2.01	.138	−0.96	6.93
		Attachment anxiety	−1.80	1.79	.316	−5.31	1.72	−3.90	1.81	.031	−7.44	−0.36
		Friends and family touch	−1.94	2.51	.439	−6.85	2.97	−1.74	2.53	.492	−6.69	3.22
		Current intimate touch	−1.47	3.11	.636	−7.57	4.62	−0.42	3.13	.894	−6.56	5.72
		Childhood touch	−0.13	1.71	.937	−3.49	3.22	−1.14	1.73	.509	−4.53	2.24
		Attitude to self-care	0.65	1.78	.714	−2.84	4.14	1.90	1.80	.290	−1.62	5.42
		Attitude to intimate touch	−0.91	4.18	.827	−9.10	7.27	−1.30	4.21	.758	−9.55	6.96
		Attitude to unfamiliar touch	4.79	2.15	.026	0.57	9.02	5.24	2.17	.016	0.99	9.50
	Intercept		0.29	14.30	.984	−27.74	28.33	−6.14	14.41	.670	−34.38	22.10
**Theta**			**Frontal electrode sites**					**Central electrode sites**				
	Predictors of interest	Country (SA is reference)	6.09	4.43	.169	−2.60	14.77	6.22	4.32	.150	−2.25	14.70
		Velocity (fast is reference)	−0.19	1.78	.914	−3.68	3.29	3.51	2.00	.079	−0.41	7.43
		Country × velocity	−4.95	2.56	.053	−9.98	0.07	−5.43	2.88	.060	−11.08	0.22
		Body region (arm is reference)	6.32	1.75	**<.001**	2.90	9.74	4.32	1.96	.028	0.47	8.16
		Country × body region	−11.36	2.54	**<.001**	−16.34	−6.38	−7.86	2.86	**.006**	−13.47	−2.26
		Velocity × body region	0.64	2.55	.803	−4.36	5.63	0.64	2.86	.823	−4.97	6.26
		Country × velocity × body region	10.03	3.65	**.006**	2.88	17.19	3.43	4.10	.404	−4.62	11.47
	Covariates	Attachment avoidance	4.13	2.51	.100	−0.78	9.04	4.28	2.39	.073	−0.39	8.96
		Attachment anxiety	−4.43	2.25	.049	−8.85	−0.02	−2.92	2.14	.172	−7.12	1.28
		Friends and family touch	3.71	3.15	.238	−2.46	9.89	4.31	2.99	.150	−1.55	10.18
		Current intimate touch	−6.46	3.90	.098	−14.11	1.19	−7.13	3.72	.055	−14.42	0.16
		Childhood touch	−1.03	2.15	.632	−5.25	3.19	0.70	2.05	.731	−3.31	4.72
		Attitude to self-care	−4.15	2.24	.063	−8.54	0.23	−6.39	2.13	**.003**	−10.56	−2.22
		Attitude to intimate touch	0.80	5.25	.878	−9.48	11.09	1.54	5.00	.757	−8.25	11.33
		Attitude to unfamiliar touch	5.99	2.71	.027	0.69	11.29	5.40	2.57	.036	0.35	10.44
	Intercept		9.23	17.96	.607	−25.97	44.43	4.90	17.13	.775	−28.68	38.48

*P* values that survived the Benjamini–Hochberg correction are highlighted in bold font.

#### Beta band

**Figure 3.**
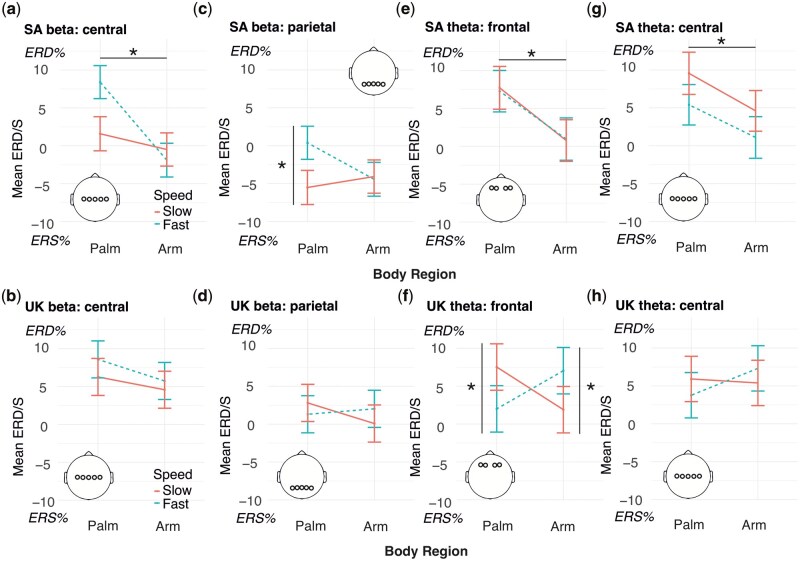
Effects of country plotted by body region (*x* axis) and velocity (separate lines; fast = dotted lines). UK: *N* = 15; SA: *N* = 19 participants. Error bars denote ±1 standard error of the mean. For central beta, the difference between palm and arm (across velocities) was significant in SA (a; planned contrast palm vs. arm = 6.19, SE = 1.0, *p* < .001) but not the UK sample (b; contrast = 2.27, SE = 1.05, *p* = .060). For parietal beta ERD/S in SA participants (c), the slow vs. fast contrast was significant only at the palm (contrast slow vs. fast = −5.92, SE = 1.57, *p* < .001) and not the arm (contrast slow vs. fast = 0.34, SE = 1.56, *p* = .999) body region, while in the UK (d), neither of these Bonferroni-corrected contrasts were significant (palm slow vs. fast contrast = 1.50, SE = 1.19, *p* = .417; arm contrast = −1.94, SE = 1.19, *p* = .204). For frontal theta, ERD was reduced for the arm compared to palm body regions in SA participants (e; frontal theta contrast = 6.64, SE = 1.24, *p* < .001) but not UK participants (f; frontal theta contrast = 0.30, SE = 1.31, *p* = .999). However, in the UK sample (f), the slow vs. fast contrast was significant at both palm (contrast = 5.52, SE = 1.75, *p* = .003) and arm (contrast = −5.15, SE = 1.75, *p* = .007) body regions, but in opposite directions, with greater ERD for slow vs. fast touch at the palm, and lower ERD for slow vs. fast touch at the arm body region. For central theta, ERD was reduced for the arm compared to palm body regions in SA participants (panel g; central theta contrast = 4.64, SE = 1.40, *p* = .002) but not UK participants (panel h; central theta contrast = −1.51, SE = 1.47, *p* = .607). Positive values correspond to event-related desynchronization (ERD); negative values correspond to event-related synchronization (ERS). Asterisks denote significant effects.

For both central and parietal electrode sites, the effects of body region were shaped by cultural context (Fig. 3a-d; marginal *R*^2^ = 0.19 and conditional *R*^2^ = 0.53 for the model involving central electrodes; marginal *R*^2^ = 0.23 and conditional *R*^2^ = 0.57 for the model involving parietal electrodes). The difference in the strength of beta-band ERD between palm and arm in bilateral central electrodes was significant in SA but not UK participants. SA participants showed larger ERD in central electrodes during touch to the palm vs. ERS during touch to the arm (see planned contrast statistics in Fig. 3a and b). Planned contrasts were not significant for beta-band ERD in parietal electrodes, though the trend was in the same direction as the central site (Fig. 3c and d). The effect in parietal electrodes was further qualified by a three-way interaction of country, velocity, and body region (see Table 4). Breaking this interaction down by country, the velocity by body region interaction for beta-band ERD/S in parietal electrodes was significant in SA (*b = *−8.27, SE = 2.24, *p =* .005) and UK participants (*b = *3.44, SE* = *1.68, *p =* .041); there was stronger ERS for slow vs. fast touch at the palm but not the arm in SA participants, whilst none of the planned contrasts were significant in the UK sample (Fig. 3c and d). These results are in line with our hypothesis that SA (vs. UK) participants would show enhanced differentiation in their neural activation patterns.

#### Theta band

There was a country by body region interaction in theta-band ERD/S in both frontal and central electrode sites (Table 4; Fig. 3e–h; marginal *R*^2^ = 0.31 and conditional *R*^2^ = 0.64 for the model involving frontal electrodes; marginal *R*^2^ = 0.27 and conditional *R*^2^ = 0.52 for the model involving central electrodes). At both sites, as for beta-band oscillations, theta-band ERD was reduced for the arm compared to the palm region in SA participants (Fig. 3e and g) but not UK participants (Fig. 3f and h), pointing to greater differentiation between body regions but not stroking speeds in SA participants. However, the three-way interaction of country by velocity by body region was also significant for the theta band in frontal electrodes. Here, the velocity by body region interaction was significant in the UK (*b *= 10.67, SE* = *2.48, *p <* .001) but not the SA sample (*b* = 0.60, SE = 2.66, *p* = .820). In the UK sample, the slow vs. fast contrast was significant at both palm and arm body regions (Fig. 3f), but in opposite directions, with larger ERD for slow vs. fast touch on the palm and lower ERD for slow vs. fast touch on the arm. While SA participants differentiated between body regions, UK participants additionally differentiated between stroking speeds, with opposite directions of effects at the arm and palm.

## Discussion

This is the first study to experimentally investigate the association of cultural context with self-reported and neurophysiological responses to affective touch, accounting for individual differences in touch experiences and attitudes. Comparing women living in SA and the UK, we found that cultural context modulated both affective (how pleasant, comfortable, liked, and wanted the touch was rated) and intensity evaluations of dynamic stroking touch, as well as cortical oscillatory patterns (ERD/S) in beta and theta bands. ERD/S in the alpha band did not differ between countries.

SA participants rated all touch more positively and less intense than did UK participants, aligning with our hypotheses based on touch norms and exposure. Also across countries, slow-velocity touch was evaluated more positively than faster touch, replicating a robust main effect in the literature (Essick *et al.* 1999, Löken *et al.* 2009). This effect was evident across both body regions (cf. Cruciani *et al.* 2021), supporting findings that top-down modulatory factors, beyond activation of CT-afferent fibres, contribute to the sensation and evaluation of affective touch (Morrison 2023). While general attitudes towards touch from intimate others, current levels of intimate touch, friends and family touch, levels of positive childhood touch, and adult attachment styles did not differ between countries, attitudes to unfamiliar touch were less positive in SA (vs. UK) participants. The discrepancy between touch ratings and self-reported attitudes may be explained by the study environment, in which touch was delivered in a controlled, arguably safe way by a trained experimenter of the same gender as participants. By contrast, attitudes to unfamiliar touch may be more generally influenced by high rates of interpersonal violence in SA (Richter *et al.* 2018).

Cortical oscillations in response to touch were modulated by cultural context in the beta and theta bands. Participants in SA but not the UK showed enhanced differentiation between touch to body regions in the beta band, with larger central ERD during touch to the palm (vs. ERS to the arm) and stronger parietal ERS for slow vs. fast touch at the palm (but not the arm), a contrast that was not significant in the UK sample. Sensorimotor beta oscillations play an active role in endogenous top-down processing and sensorimotor integration by linking sensory input with contextual knowledge (Barone and Rossiter 2021). Pre-stimulus beta oscillations in the primary somatosensory cortex are influenced by tactile expectations, with this effect enhanced by attention (van Ede *et al.* 2010). Moreover, beta oscillations correspond to somatosensory decision outcomes, responding selectively to stimulus features only when they are task-relevant (Herding *et al.* 2016). Taken together, enhanced desynchronization of central beta oscillations during fast touch and touch to the palm in participants in SA may represent context-specific endogenous modulation of neural sensory processing, potentially shaped by cultural differences in touch experiences and expectations, as we explore further below.

Theta oscillations were also modulated by cultural context, possibly reflecting differences in the social or emotional importance of touch. UK participants showed lower frontal ERD for slow (vs. fast) touch to the arm. This finding is opposite to prior research pointing towards a potentially soothing effect of pleasant and prosocial touch, denoted by lower absolute theta power across the scalp in response to slow, CT-optimal touch (compared to faster, CT-suboptimal touch; von Mohr *et al.* 2018a), and in frontal sites in response to static, supportive hand-holding from a partner (Kraus *et al.* 2020) compared to no touch or hand-holding from a stranger. This discrepancy may stem from methodological differences between absolute band power changes (von Mohr *et al.* 2018a, Kraus *et al.* 2020) and power change relative to baseline in the current study. Our findings may reflect heightened theta power during the preceding baseline period, which diminished during touch. This relative increase aligns with studies linking theta synchronization to emotion regulation (Ertl *et al.* 2013), suggesting greater regulatory engagement during slow (vs. fast) arm touch in UK participants. Additionally, UK participants also showed lower ERD for fast (vs. slow) touch to the palm. As frontal theta ERS is linked with somatosensory orienting (Dietl *et al.* 1999), attention, and cognitive control (Cavanagh and Frank 2014), lower ERD for fast (vs. slow) palm stroking in UK participants suggests enhanced engagement of top-down attentional processes. This aligns with evidence that faster stroking touch is associated with communicating intentions of warning (Kirsch *et al.* 2018), potentially eliciting heightened attention. However, as the oscillatory changes that we observed were during continuous brushing rather than at brushing onset (see Supplementary Material Fig. 4), changes in relative theta power in UK participants are unlikely to relate to differences in early sensory or orienting responses, typically reflected by phasic theta ERS. Furthermore, as theta research has largely focused on ERS, the underlying mechanisms of theta ERD during affective touch have yet to be determined. Given the lack of prior studies examining cortical oscillations in response to dynamic palm touch, future research is needed to clarify its functional significance.

In contrast to UK participants, SA participants exhibited reduced frontal and central theta-band ERD (less theta suppression) for arm versus palm touch across speeds. When considered alongside beta-band results, this suggests greater significance of palm touch in SA participants compared to those in the UK. The relevance of touch to the palm in SA participants may extend beyond sensory attributes to social and cultural dimensions. Previous research has found differences in touch permissibility to different body sites as a function of emotional closeness e.g. stranger touch is more acceptable to the hand than to the forearm (Suvilehto *et al.* 2015), and there are cultural differences in terms of the strength of this association for arm vs. hand in British vs. Japanese cultural contexts (Suvilehto *et al.* 2019). Moreover, Chinese participants preferred touch to the hands more than did German participants (Schirmer *et al.* 2023). The arm and hand may therefore hold different significance in different cultural contexts, and further research is needed to investigate our effect of heightened cortical engagement in relation to palm vs. arm touch in participants living in SA. In SA, palm touch may hold augmented social significance, but this remains to be fully explored.

Alpha band power showed consistent effects across countries. Faster touch induced stronger central alpha ERD, and touch applied to the palm was associated with greater central alpha ERD and reduced parietal alpha ERS compared to the arm; 10 and 20 Hz ERD in somatosensory regions have been associated with cortical excitability and readiness for sensory processing (Stančák 2006), as well as attentional resource allocation and arousal (Neuper and Pfurtscheller 2001). Thus, augmented cortical oscillations may be driven more by bottom-up stimulus properties. Heightened cortical activation signified by stronger ERD for palm touch might be linked to the palm’s greater innervation density and smaller receptive fields (Vallbo *et al.* 1995) and greater tactile acuity (Ackerley *et al.* 2014a) compared to the forearm. These features facilitate precise sensory processing, especially during tasks requiring detailed mapping of stimulus characteristics, such as object manipulation and grasping (Johansson and Vallbo 1983). The hands have larger cortical somatotopic representations compared to the forearms and much of the rest of the body, further emphasizing their importance in detailed sensory processing (Penfield and Rasmussen 1950). Sensory-driven processes are further shaped by social factors, with increased alpha power over frontal and parietal regions found when participants were at rest with somebody else present vs. at rest alone (Verbeke *et al.* 2014). In sum, alpha-band oscillations were not modulated by cultural context, indicating that while potentially shaped by individual differences, these attentional processes may not be culturally specific.

A strength of our study was investigating not only cross-cultural differences but also measuring individual differences relating to touch attitudes and experiences, and controlling for inter-individual variance in analyses. Thus, our findings are not due to e.g. differences in individual attachment styles or current intimate touch levels between participants in the two countries. Moreover, we asked participants to rate touch in relation to five aspects (pleasantness, comfort, intensity, liking, and wanting) and found that the four affective terms formed an internally consistent scale. In future, researchers may choose to include one or more of these aspects that seem to relate to similar evaluative constructs, at least at the self-report level.

Several limitations regarding the broad use of ‘culture’ and sample representativeness should be noted. Our samples are not representative of the entire populations living in SA and the UK, nor were all our participants South African or British nationals. Both SA and the UK are home to individuals from various countries and ethnic backgrounds, which likely influence their touch norms and experiences. Moreover, early touch experiences might be different for people who moved to SA or the UK in adulthood. Future studies should explore specific cultural groups within these countries to investigate more nuanced cross-cultural differences in affective touch experiences. The influence of sociodemographic factors such as religiosity (e.g. as in Sorokowska *et al.* 2021) on affective touch perception (both within and between cultural contexts) also warrants further investigation. Measures such as attachment style, rooted in Western constructs (Keller 2018), may also not be the most appropriate way of assessing mental representations of social relationships across cultural contexts. Nevertheless, experimental studies that draw on neuroimaging methods within cognitive neuroscience are rare and challenging to conduct in contexts such as SA (see Besharati and Akinyemi 2023, Cockcroft 2025), resulting in further underrepresentation of African populations in EEG and experimental neuroscience research (Kwasa 2024). Thus, the current study is a critical step in adding to the diversity and representation of majority-world participants in cognitive neuroscience generally and in affective touch research more specifically.

Although we hypothesized that cultural context may underlie cortical oscillatory changes during affective touch, we acknowledge that methodological differences between sites, including differences in EEG systems and laboratory environments, may have also contributed to the observed findings. Therefore, while we suggest cultural factors are important, the impact of these methodological variations should also be considered when interpreting the findings. However, previous work suggests that individual participant differences account for substantially more variance in EEG data compared to hardware differences. For instance, Melnik *et al.* (2017) reported that between-subject variability explained 32% of the variance in event-related potentials, while different EEG systems accounted for only 9%. These findings support the robustness of cross-system comparisons when appropriate harmonization procedures are in place. Standardized analysis pipelines, as in the current study, have also been found to reduce variability in multi-lab EEG studies (Farzan *et al.* 2017). We also acknowledge that environmental differences between laboratories—such as lighting conditions, ambient noise, and other contextual factors—may have influenced participant arousal or comfort. However, such variation is inherent and widely recognized as a challenge in both multi-site and cross-cultural research, where the use of different labs is not only unavoidable but essential (see Pavlov *et al.* 2021). This issue is similarly present in wider neuroimaging research, where data are frequently acquired (and compared) on different MRI systems across sites (Warrington *et al.* 2025).

Further methodological limitations should also be considered. Although our sample size aligns with prior EEG studies of affective touch (Ackerley *et al.* 2013, von Mohr *et al.* 2018a, Haggarty *et al.* 2020), it remains relatively modest. A formal power analysis was not conducted due to the multidimensional nature of the data and practical constraints associated with multi-site EEG research conducted in resource-limited settings. Moreover, as this study included only women, the findings may not generalize to other genders. While the current findings provide a valuable foundation, future studies should aim to replicate and extend these results in larger, more representative samples across both the UK and SA contexts. Finally, while care was taken to standardize touch delivery across sites using training and auditory cues, manual brushing introduces minor variability in brush speed and pressure. This method aligns with validated procedures in affective touch research, and small variations are unlikely to systematically impact outcomes due to the broad tuning of CT afferents (Löken *et al.* 2009). Future studies could consider using robotic tactile stimulators to further enhance precision, though such methods may reduce the naturalistic qualities of human social touch.

In conclusion, our study provides the first experimental and neuroimaging evidence showing that cultural context modulates both subjective and neurophysiological responses to affective touch. We observed effects of cultural context on touch evaluation across body region and velocity. The neural data paint a more nuanced picture, suggesting there may be a disconnect between cortical representations and subjective touch evaluations. Taken together, extrapolating findings from a given study to populations in a different cultural context is not appropriate. Given the increasing use of touch in therapeutic contexts (McGlone *et al.* 2024), indications of cross-cultural differences in health benefits as a function of affective touch (Packheiser *et al.* 2024), and the cultural diversity within and between countries in an increasingly globalized world, we strongly recommend that researchers developing and implementing touch-based interventions (cf. Packheiser *et al.* 2024) consider such cultural factors as well as individual differences in their design and in the interpretation of their findings.

## Supplementary Material

nsaf082_Supplementary_Data

## Data Availability

Source data and code are available on the Open Science Framework: https://osf.io/fcqnk.
